# Autonomous chest x-ray image classification, capabilities and prospects: rapid evidence assessment

**DOI:** 10.3389/fdgth.2025.1685771

**Published:** 2026-01-13

**Authors:** Yuriy Vasilev, Alexander Bazhin, Roman Reshetnikov, Olga Nanova, Anton Vladzymyrskyy, Kirill Arzamasov, Pavel Gelezhe, Olga Omelyanskaya

**Affiliations:** 1 Research and Practical Clinical Center for Diagnostics and Telemedicine Technologies of the Moscow Health Care Department, Moscow, Russia; 2 National Medical and Surgical Center Named After N.I. Pirogov of the Ministry of Health of the Russian Federation, Moscow, Russia; 3 I.M. Sechenov First Moscow State Medical University of the Ministry of Health of the Russian Federation, Sechenov University, Moscow, Russia; 4 Ministry of Science and Higher Education, Moscow Technical University—MIREA, Moscow, Russia

**Keywords:** AI, autonomous triage, chest x-ray, radiology, screening

## Abstract

**Background:**

Screening methods are essential for detection of numerous pathologies. Chest x-ray radiography (CXR) is the most widely used screening modality. During the screening, radiologists primarily examine normal radiographs, which results in a substantial workload and an increased risk of errors. There is an increasing necessity to automate radiological screening in order to facilitate the autonomous sorting of normal studies.

**Objective:**

We aimed to evaluate the capabilities of artificial intelligence (AI) techniques for the autonomous CXRs triage and to assess their potential for integration into routine clinical workflow.

**Methods:**

A rapid evidence assessment methodology was employed to conduct this review. Literature searches were performed using relevant keywords across PubMed, arXiv, medRxiv, Elibrary, and Google Scholar covering the period from 2019 to 2025. Inclusion criteria comprised large-scale studies addressing multiple pathologies and providing abstracts in English. Meta-analysis was conducted using confusion matrices derived from reported diagnostic performance metrics in the selected studies. Methodological quality and the overall quality of evidence were assessed using a combination of QUADAS-2, QUADAS-CAD, and GRADE frameworks.

**Results:**

Out of 327 records, 11 studies met the inclusion criteria. Among these, three studies analyzed datasets reflecting the real-world prevalence of pathologies. Three studies included very large cohorts exceeding 500,000 CXRs, whereas the remaining studies used considerably smaller samples. The proportion of autonomously triaged CXRs ranged from 15.0% to 99.8%, with a weighted average of 42.3% across all publications. Notably, in a study conducted under real-world clinical conditions on continuous data flow, this proportion was 54.8%. Sensitivity was 97.8% (95% CI: 94.8%–99.1%), and specificity was 94.8% (95% CI: 53.0%–99.7%). Fifty-five percent of the studies were classified as having a low risk of bias. Primarily, elevated risk of bias and heterogeneity of results were attributed to variability in sample selection criteria and reference standard evaluation.

**Conclusions:**

Modern AI systems for autonomous triage of CXRs are ready to be implemented in clinical practice. AI-driven screening can reduce radiologists' workload, decrease sorting errors and lower the costs associated with screening programs. However, implementation is often hindered by regulatory and legislative barriers. Consequently, comprehensive clinical trials conducted under real-world conditions remain scarce.

## 1 Introduction

### 1.1 Rationale

Chest radiography is one of the most frequently performed preventive radiological examinations. However, the ongoing shortage of radiologists (1–4) and the subsequent adoption of new x-ray equipment, makes the optimization of radiological workflows for screening studies increasingly critical. The vast majority of these preventive diagnostic imaging studies reveal an absence of pathological findings. The reported prevalence of pathologies in chest radiographs ranges from 1% to 20% (5–8). This low prevalence implies that radiologists spend most of their time interpreting “normal” examinations, which may inadvertently increase the risk of missing abnormalities. This phenomenon is an example of cognitive bias, where a radiologist, upon detecting an initial finding or confirming the absence of pathology, prematurely terminates the image analysis. Several factors contribute to such diagnostic errors, including «satisfaction of search», «localisation errors», «lack of thorough structural analysis», and «data shortage» (9).

In order to mitigate the risk of missed pathologies and reduce the workload of radiologists, clinical decision support systems—particularly those employing artificial intelligence (AI)—have gained widespread adoption. In the context of screening examinations, a notable approach is the autonomous triage of chest radiographs (CXRs), whereby AI algorithms differentiate normal from abnormal images without direct radiologist involvement. In practice, this means the automated exclusion of routine CXRs, enabling radiologists to concentrate on more complex cases. The primary objective of autonomous triage is to alleviate the burden on radiologists and reduce reporting delays while maintaining patient safety (10, 11). Over the past five years, numerous studies have investigated AI systems for autonomous chest radiograph triage.

### 1.2 Objectives

The present study aimed to evaluate the capabilities of contemporary AI methods for the autonomous triage of CXRs and assess their prospects for integration into routine clinical practice. Although numerous publications have explored AI for the detection and screening of individual diseases, such narrowly focused strategies have limited applicability in practical screening workflows.

In this study, we conducted a selective review of scientific literature to determine the performance and applicability of modern AI for autonomous chest radiograph triage. We employed a Rapid Evidence Assessment (REA) (12) methodology to enable a rapid yet comprehensive evaluation of the feasibility and clinical utility of autonomous triage systems in current real-world practice.

## 2 Methods

The prospective, unregistered study protocol is provided in the Supplementary Material.

### 2.1 Literature search

The article search was conducted for the period from 2019 to 2025. All articles and preprints with an English abstract were considered. Searches were performed across multiple databases: PubMed, arXiv, medRxiv, Elibrary. An additional literature search was conducted using the Google Scholar search engine.

The search query used in PubMed was as follows:

(“Radiography, Thoracic”[Mesh] OR Thoracic Radiograph*[tiab] OR (x-ray[tiab] AND (chest[tiab] OR thora*[tiab])) OR CXR) AND (“Radiographic Image Interpretation, Computer-Assisted”[Mesh] OR Artificial Intelligence[tiab] OR Computer vision[tiab] OR (learning[tiab] AND (machine[tiab] OR deep[tiab]))) AND (Autonom*[tiab] OR standalone[tiab] OR unaided[tiab] OR unassisted[tiab]).

The search query for arXiv was: “Autonomous AI AND chest x-ray OR CXR AND triage”. The search query for medRxiv, Elibrary, and Google Scholar was: “Autonomous AI chest x-ray triage”.

### 2.2 Study selection

The present study incorporated all screening studies that used CXRs to analyze multiple pathologies. A two-stage approach was employed for study selection. In the initial phase, two independent reviewers screened titles and abstracts to identify the most relevant studies to our research question. In the subsequent stage, the full texts of articles that passed the initial screening were independently reviewed by two researchers, who made inclusion decisions.

The search and selection of articles were carried out by two researchers, each with over 10 years of experience in medical informatics.

### 2.3 Data extraction

The following information was extracted from the selected studies: bibliometric data, country of study, research objectives, study design (prospective/retrospective, multicenter/single-center, binary classification/prioritization), number of abnormalities, employed AI algorithm, inclusion and exclusion criteria, reference standard, assessment of time benefits and economic efficiency, all values of AI diagnostic accuracy, values of true positives (TP), false negatives (FN), false positives (FP), true negatives (TN), and comparison of AI diagnostic accuracy with that of physicians.

Information extraction was conducted independently by two researchers, each with over 10 years of experience in medical informatics.

### 2.4 Meta-analysis

The number of included and autonomously triaged CXRs for each study was reported.

Components of the confusion matrix that were not reported in the publications were independently calculated based on the reported diagnostic performance metrics, following the methodology suggested by (13). In studies employing prioritization, the category designated as “normal” was classified as normal, whereas all other categories (“non-urgent”, “urgent”, and “critical”) were classified as abnormal. Subsequently, sensitivities and specificities were calculated for each study with corresponding 95% confidence intervals (CIs). Paired forest plots displayed individual study estimates alongside pooled meta-analysis results for sensitivity and specificity. Additionally, a receiver operating characteristic (ROC) plot of sensitivity vs. 1-specificity summarized diagnostic performance based on meta-analysis data.

The meta-analysis was conducted using the MetaDTA tool (14).

The expected prevalence level of abnormalities was calculated per 10,000 cases based on minimal, maximal, and real-world findings in the selected studies.

A sensitivity analysis was conducted across two scenarios. In the first, two studies employing prioritization were excluded from the overall sample. The second scenario excluded the two studies with the highest sensitivity and specificity values (occupying the upper left corner on the ROC plot) from the overall sample.

Subgroup and random effects analyses were conducted to investigate potential sources of heterogeneity. The analyses were performed using the *metafor* package for R, employing the REML (Restricted Maximum Likelihood) model for small samples. The factors used were sample size (as a continuous variable), year of study publication (as a continuous variable), and population (as a discrete binary variable: general or hospital).

### 2.5 Assessment of methodological quality

Two review authors independently assessed the methodological quality of included studies using a combination of two instruments: QUADAS-2 (15) and the AI-adapted QUADAS-CAD (16, 17). We assessed each of the four domains (Patient Selection, Index Test, Reference Standard, Flow and Timing) in terms of risk of bias using QUADAS-CAD, and the first three domains in terms of concerns regarding applicability to the review question using QUADAS-2.

Any disagreements were resolved through discussion to reach consensus. If needed, a third author facilitated consensus.

In the Patient Selection domain, we assessed whether the screening samples reflected the distribution of normal and pathological cases in the real-world population. In the Index Test domain, we analyzed how the index test was applied. In the Reference Standard domain, we evaluated the types of reference standards used, taking into account the presence of multiple pathologies in the samples. In the Flow and Timing domain, we assessed whether the reference standards were consistently applied across all cases and examined the overall transparency of the results.

We visually examined forest plots and ROC plots for heterogeneity and discussed potential data heterogeneity sources. These estimations were further used in the assessment of certainty of evidence. We assessed the certainty of evidence using the GRADE methodology (18, 19).

## 3 Results

### 3.1 Results of the search and study selection

**Figure 1:**
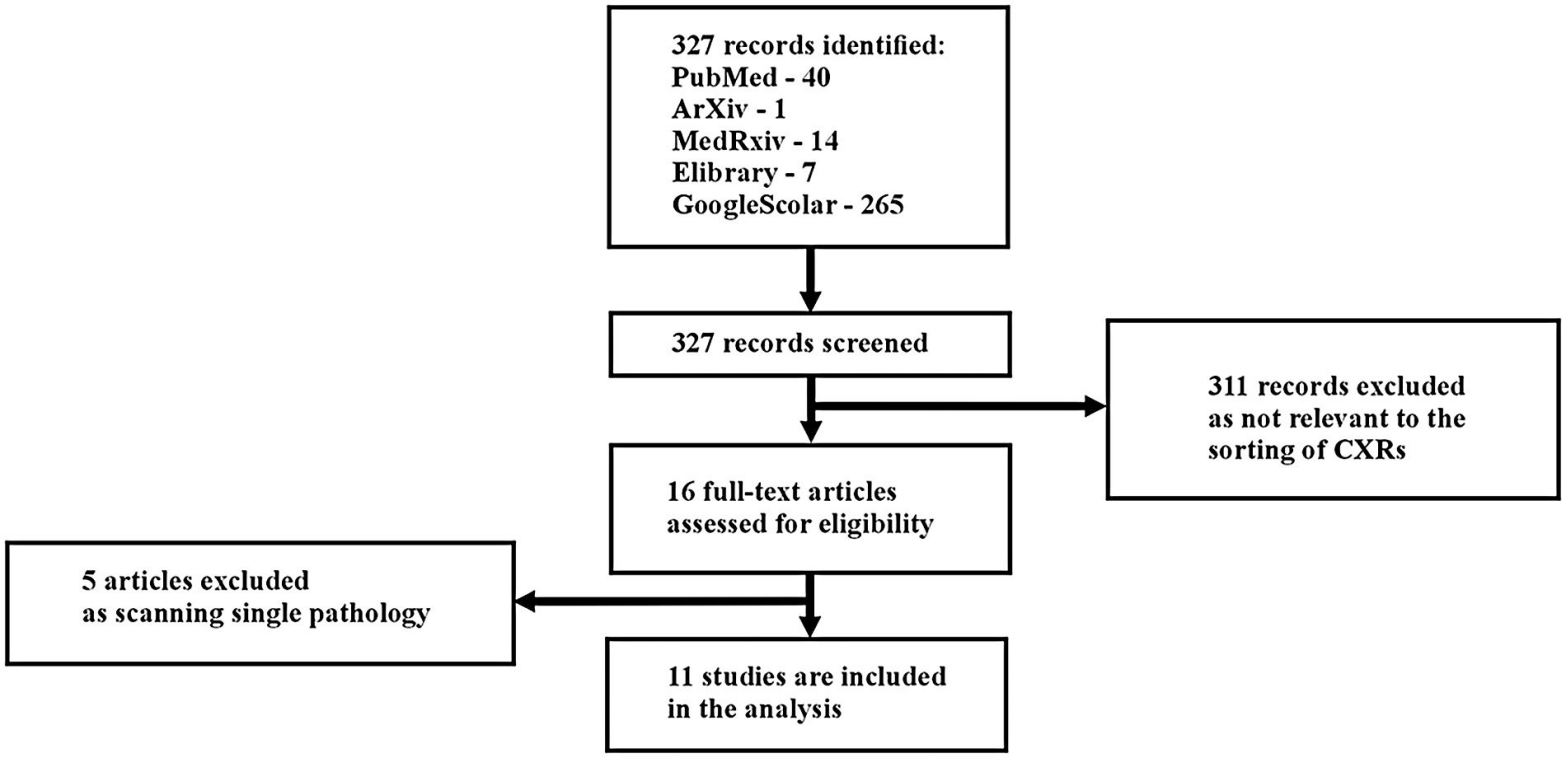
Study flow diagram.

327 records were identified (Figure 1). Of these, 40 records were identified in PubMed, 1 in arXiv, 14 in medRxiv, 7 in Elibrary, and 265 in Google Scholar.

311 records were excluded as irrelevant to the classification of CXRs. After the initial screening, 16 articles underwent full-text analysis: 5 from PubMed, 1 from arXiv, 0 from medRxiv, 1 from Elibrary, and 9 from Google Scholar.

During the full-text review, 5 articles were excluded because they focused on the detection of a single pathology (Appendix 1). Consequently, 11 articles were included in the systematic review: 3 from PubMed, 1 from arXiv, 0 from medRxiv, 1 from Elibrary, and 6 from Google Scholar.

In the study by (20), several distinct datasets were employed. For inclusion in our review, we selected two datasets relevant to our research focus (DS-1 and CXR-14), which involved multiple pathologies, and excluded model datasets containing only a single target pathology (e.g., tuberculosis or COVID-19).

Confusion matrices were reported in two publications (10, 21). For the remaining studies, we independently calculated confusion matrices based on the reported diagnostic performance metrics, following the methodology described in (13).

### 3.2 Main characteristics of the studies

The main characteristics of the selected publications are presented in Table 1 (general study information) and Table 2 (quantitative characteristics).

**Table 1:** The studied literature.

№	First author/year	Title	Journal	Location
1	Vasilev et al. (22)	Autonomous artificial intelligence for sorting results of preventive radiological examinations of chest organs: medical and economic efficiency	Digital Diagnostics	Russian Federation
2	Blake et al. (11)	Using Artificial Intelligence to stratify normal versus abnormal Chest x-rays: external validation of a deep learning algorithm at East Kent Hospitals University NHS Foundation Trust	Diagnostics	United Kingdom
3	AlJasmi et al. (28)	Post-deployment performance of a deep learning algorithm for normal and abnormal chest x-ray classification: A study at visa screening centers in the United Arab Emirates	European Journal of Radiology Open	United Arab Emirates
4	Plesner et al. (10)	Autonomous chest radiograph reporting using AI: estimation of clinical impact	Radiology	Denmark
5	Yoo et al. (24)	Artificial Intelligence-Based Identification of Normal Chest Radiographs: A Simulation Study in a Multicenter Health Screening Cohort	Korean J Radiology	Republic of Korea
6	Schalekamp et al. (26)	Performance of AI to exclude normal chest radiographs to reduce radiologists' workload	European Radiology	The Netherlands
7	Subramanian et al. (25)	Autonomous AI for multi-pathology detection in Chest x-Rays: a multi-site study in Indian healthcare system	arXiv	Republic of India
8	Sridharan et al. (23)	Real-World evaluation of an AI triaging system for chest x-rays: A prospective clinical study	European Journal of Radiology	Singapore
9	Annarumma et al. (21)	Automated triaging of adult Chest Radiographs with Deep Artificial Neural Networks	Radiology	United Kingdom
10	Dyer et al. (27)	Diagnosis of normal chest radiographs using an autonomous deep-learning algorithm	Clinical Radiology	United Kingdom
11	Nabulsi et al. (20)	Deep learning for distinguishing normal versus abnormal chest radiographs and generalization to two unseen diseases tuberculosis and COVID-19	Scientific Reports	USA

№	Objective	Design	Abnormalities number	AI algorithms
1	To evaluate the feasibility, effectiveness, and efficiency of autonomous sorting of results from preventive radiological examinations of chest organs	Prospective, multicenter, binary classification	9	The experiment used medical products based on AI technologies to analyse the results of CXRs and fluorography: – PhthisisBioMed Artificial Medical Intelligence: Software for Automated Analysis of Digital Chest x-ray/Fluorograms (medical device registration certificate No. RZN 2022/17406 dated May 31, 2022) [10.17691/stm2023.15.4.01]; – «Third Opinion. Fluorograms/Chest x-ray » (ООО «Third Opinion Platform»; No. RZN 2021/14506); – «CELSUS» (ООО «Medical screening systems»; No. RZN 2021/14449)
2	To evaluate the performance and clinical utility of a fully automated computer-aided detection system in stratifying CXRs as normal or abnormal as well as its potential impact on optimizing the workload of reporting radiologists while ensuring patient safety	Retrospective, multicenter, binary classification	Unknown: various abnormal findings	qXR (CE-marked)
3	To assess the agreement of AI in classifying normal versus abnormal CXRs with reporting radiologists, to assess clinical and technical users provide insights into the perceived implementation challenges and benefits of integrating AI into clinical workflows	Retrospective, multicenter, binary classification	Unknown: multiple abnormalities	qXR version 2.1 (CE-marked)
4	To perform an external evaluation of a commercially available AI tool for (a) the number of chest radiographs autonomously reported, (b) the sensitivity for AI detection of abnormal chest radiographs, and (c) the performance of AI compared with that of the clinical radiology reports	Retrospective, multicenter, binary classification	38	ChestLink, version 2.6 (CE-marked); Oxipit
5	To investigate the feasibility of using AI to identify normal CXR from the worklist of radiologists in a health-screening environment	Retrospective, multicenter, binary classification	10	Lunit INSIGHT CXR3, version 3.5.8.8 (CE mark, FDA)
6	To investigate the performance of a commercially available AI to identify normal CXRs and its potential to reduce radiologist workload	Retrospective, multicenter, binary classification	10	Lunit INSIGHT CXR3 (CE mark, FDA)
7	To study AI-based approach capabilities to CXRs pathology detection in the Indian healthcare system	Retrospective, multicenter, binary classification	75	This AI system integrates advanced architectures, including Vision Transformers, Faster R-CNN, and various U-Net models (such as Attention U-Net, U-Net++, and Dense U-Net)
8	To evaluate AI triaging software performance in a prospective, real-world setting	Prospective, single center, prioritization	Unknown	LUNIT INSIGHT CXR Triage (CE mark, FDA)
9	To develop and test an AI system, based on deep convolutional neural networks, for automated real-time triaging of adult CXRs on the basis of the urgency of imaging appearances	Retrospective, multicenter, prioritization	15	Ensemble of two CNNs
10	To evaluate the suitability of a deep-learning algorithm for identifying normality as a rule-out test for fully automated diagnosis in frontal adult CXRs in an active clinical pathway	Retrospective, multicenter, binary classification	Unknown	The ensemble contained a mix of two different types of state-of-the-art architectures, Densenet121 and EfficientNet B4
11	To develop deep learning system that classifies CXRs as normal or abnormal using data containing a diverse array of CXR abnormalities from 5 clusters of hospitals from 5 cities in India, to evaluate the deep learning system for its generalization to unseen data sources and unseen diseases using 6 independent datasets	Retrospective, multicenter, binary classification	Unknown	CNN

№	Inclusion criteria	Exclusion criteria	Reference test	Time benefits assessement	Economic efficiency assessment
1	Patients ≥ 18 y. o. Appointment of a preventive examination of the chest organs Radiography or fluorography of the chest organs The signed voluntary informed consent of the patient Time period: 01.05.2024–30.09.2024	Absence of results of preventive examination in Unified medical information and analytical system of the city of Moscow Technical defects in performing preventive examination	Reference test No. 1: protocol prepared by a radiologist of the Russian Medical Academy of Continuous Professional Education of the Ministry of Health of the Russian Federation Reference test No. 2: expert review by a radiologist with subspecialisation in thoracic radiology of the Scientific and Practical Clinical Center for Diagnostics and Telemedicine Technologies of the Moscow Department of Health	no	yes
2	Patients ≥ 18 y. o. PA/AP view Minimum image resolution of 1440 × 1440 Minimum of 10 gray levels	Lateral CXRs Incomplete view of the chest CXR images containing excessive motion artifacts	The agreement between two senior radiologists with experience of 10 and 12 years	no	no
3	All the consecutive frontal (PA/AP) CXRs acquired from patients ≥ aged 18 y. o. Time period: January 2021–June 2022	Any CXRs with a non-available AI result and CXRs where the radiologist suggested a repeat CXR due to lack of image quality	17 radiologists and 3 health professionals (PACS/ IT managers) who had prior experience in using the AI software	no	no
4	All patients ≥ 18 y. o. who underwent chest radiography in the hospitals, including emergency department patients, in-hospital patients, and outpatients. Only the first CXR was included, but follow-up CXRs were allowed if a primary CXRhad been acquired before the inclusion period Time period: 1–12 January 2020	Patients with duplicate records, missing Digital Imaging and Communications in Medicine, or DICOM, images, CXRs from a nontarget hospital, and/or insufficiently visualized chest	The reference standard was provided independently by two board-certified thoracic radiologists, with a third radiologist in cases of disagreement	no	no
5	Patients who visited health screening center and underwent both CXR and chest CT beween January and December 2018	Interval between CXR and chest CT was more than 1 months	Two stages: 1)Three board-certified thoracic radiologists (with 19, 13, and 12 years of experience in thoracic imaging, respectively); 2) relevant diagnosis from the electronic medical record	no	no
6	All consecutive CXRs of inpatient and outpatient collected from an academic hospital (Radboudumc, Nijmegen, The Netherlands) and a large community hospital (Jeroen Bosch Hospital, 's Hertogenbosch, The Netherlands) Emergency CXRs Only the first initial exam in inclusion period for each patient Time period: 1–14 October 2016	Bedside radiographs, pediatric and incomplete visualized chests, as well as the radiographs imported from other hospitals	3 chest radiologists (9, 12, and 20 years of experience)	no	no
7	Unclear	Unclear	Unclear	no	no
8	Underwent a CXR during the study period: August–December 2023 Above 10 years of age, given that pediatric radiology involves different considerations Had no prior CXRs taken within the same hospitalization to ensure independent assessments	Unclear	Unclear	yes	no
9	Frontal CXRs obtained from January 2005 to May 2017 at a publicly funded university hospital network consisting of three hospitals	Pediatric CXRs obtained in patients <16 y. o.	Unclear	no	no
10	All frontal CXRs performed on patients ≥ 18 y. o. Computed or digital radiography Patient type as one of accident and emergency, general practitioner or outpatient Time period: 22.12 2017–14.05.2020	Non-CXR examinations including lateral CXR and chesteabdomen radiographs	Labelling of two independent FRCR-trained radiologists, with a minimum of 10 years NHS clinical experience at consultant level. In case of a discrepancy, images and conflicting radiologists' labels were sent for arbitration by a third FRCR radiologist	no	no
11	The first dataset DS-1 was from five clusters of hospitals across five different cities in India (Bangalore, Bhubaneswar, Chennai, Hyderabad, and New Delhi)5. DS-1 consisted of images from consecutive inpatient and outpatient encounters between November 2010 and January 2018, and reflected the natural population incidence of the abnormalities in the populations The second dataset CXR-14 contains 2000 randomly selected CXRs from the publicly specified test set (25,596 CXRs from 2797 patients) of CXR-14 from the National Institute of Health	Unclear	Normal/abnormal based on majority vote of 3 radiologists	no	no

**Table 2:** Quantitative characteristics of the studies.

Study ID	Population	Total number of CXRs	Number (%) of autonomously sorted CXRs	TP	FN	FP	TN	Sensitivity %	Specificity %
Vasilev et al. (22)	General population	575,549	54.8	260,058	290	0	315,201	99.9	100.0
Blake et al. (11)	Multiple clinical patients (general practices, accident and emergency departments, and inpatient and outpatient)	993	39.3	497	3	105	387	99.4	78.7
AlJasmi et al. (28)	Migrants in screening visa centers	1,309,443	71.5	18,883	749	354,308	935,503	96.2	72.5
Plesner et al. (10)	Multiple hospitals patients including isolated areas of interstitial lung disease and lung cancer	1529	28.0	1090	10	309	120	99.1	28.0
Yoo et al. (24)	General population	5,887	42.9	632	18	2,729	2,508	97.2	47.9
Schalekamp et al. (26)	Multiple hospitals patients (inpatient, outpatient, and emergency chest radiographs)	1,670	53.0	207	18	578	867	92.0	60.0
Subramanian et al. (25)	General population	1,012,851	99.8	2,022	1,011	4	1 009 814	66.7	100.0
Sridharan et al. (23)	Patients of general hospital	20,944	28.6	12 412	299	2,542	5,690	97.6	69.1
Annarumma et al. (21)	Multiple hospitals patients	3,229	20.3	2,524	147	160	398	94.5	71.3
Dyer et al. (27)	Multiple hospitals patients (accident and emergency, general practitioner or outpatient)	3,887	15.0	3,294	13	10	570	99.6	98.3
Nabulsi et al. (20)(a) (DS-1)	Multiple hospitals patients (inpatients and outpatients)	7,747	29.9	1,792	46	3,639	2,270	97.5	38.4
Nabulsi et al. (20)(b) CXR-14	Multiple hospitals patients	810	24.0	548	29	68	165	95.0	070.8

TP, true positive; FN, false negative; FP, false positive; TN, true negative.

Only two of the 11 studies were prospective and conducted on streaming data (22, 23). Among these, only one large-scale study was performed under real-world conditions (22). Of the 11 publications, 10 were multicenter studies, and one was a single-center study (23).

Real-world population data were employed in three studies (22, 24, 25). In other cases, normal/abnormal distribution in employed samples could differ from general population: patients from hospitals (10, 11, 20, 21, 23, 26, 27) including those specializing in pulmonary pathologies (10), as well as migrant populations (28).

Most studies (9 out of 11) employed binary classification distinguishing between normal and abnormal categories. The abnormal category predominantly encompassed multiple pathologies (ranging from 9 to 75 categories) (10, 11, 22, 24–26, 28), while in some studies, the number of pathologies was unspecified (20, 27).

Two studies utilized prioritization schemes: normal, non-urgent, and urgent (23), or normal, non-urgent, urgent, and critical (21).

Most studies included CXRs of adult patients; however, two studies (23, 25) also included CXRs of patients under 18 years of age.

The largest datasets, each comprising over half a million samples, were employed in three publications (28): with 1,309,443 CXRs (accounting for 44.5% of the total review sample) (25), with 1,012,851 CXRs (34.4%), and (22) with 575,549 CXRs (19.5%).

The sample sizes of the remaining publications (10, 11, 20, 21, 23, 24, 26, 27) were considerably smaller, ranging from 810 to 20,944 CXRs (contributing from 0.03% to 0.71%, respectively).

### 3.3 Meta-analysis

The proportion of autonomously triaged CXRs varied substantially across studies, ranging from 15.0% to 99.8% (Table 2). In the largest screening studies, the number of autonomously triaged CXRs was notably high. For instance, in a real-world data stream study by (22) the proportion of autonomously triaged CXRs was 54.8%. In retrospective studies with sample sizes exceeding one million (25, 28), the proportion reached 99.8% and 71.5%, respectively.

Estimates of sensitivity and specificity are presented and summarized in Table 2. Sensitivity was consistently high across all studies, with a median value of 97.8% (95% CI: 94.8%–99.1%). Specificity was also generally high on average but exhibited considerable variability, with a median of 94.8% (95% CI: 53.0%–99.7%). In the first sensitivity analysis scenario, the median sensitivity was 97.8% (95% CI: 94.8–99.1%), and the median specificity was 94.8% (95% CI: 53.0%–99.7%). In the second scenario, the median sensitivity was 97.6% (95% CI: 93.5%–99.1%), and the median specificity was 97.5% (95% CI: 61.7%–99.9%).

**Figure 2:**
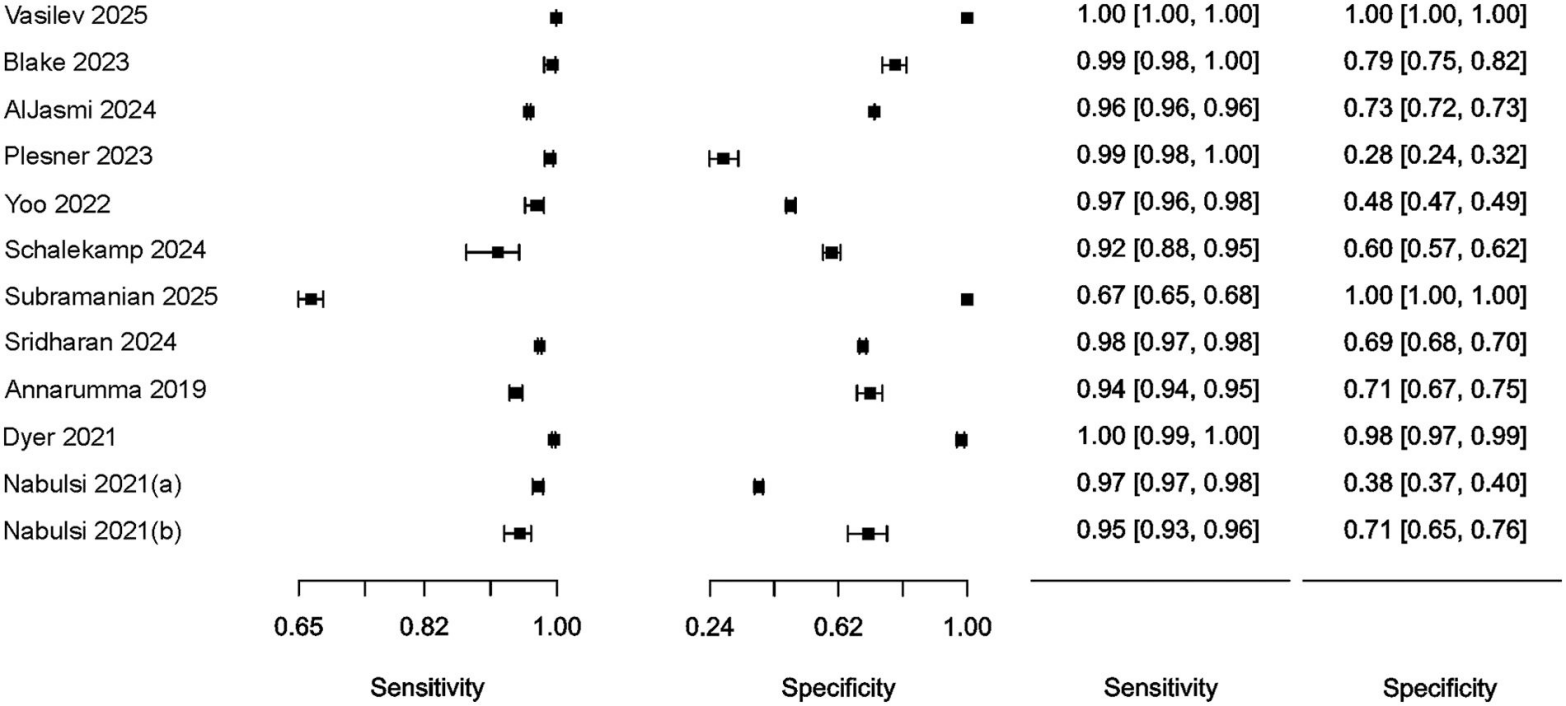
Forest plot of studies, 95% CI provided in parentheses.

**Figure 3:**
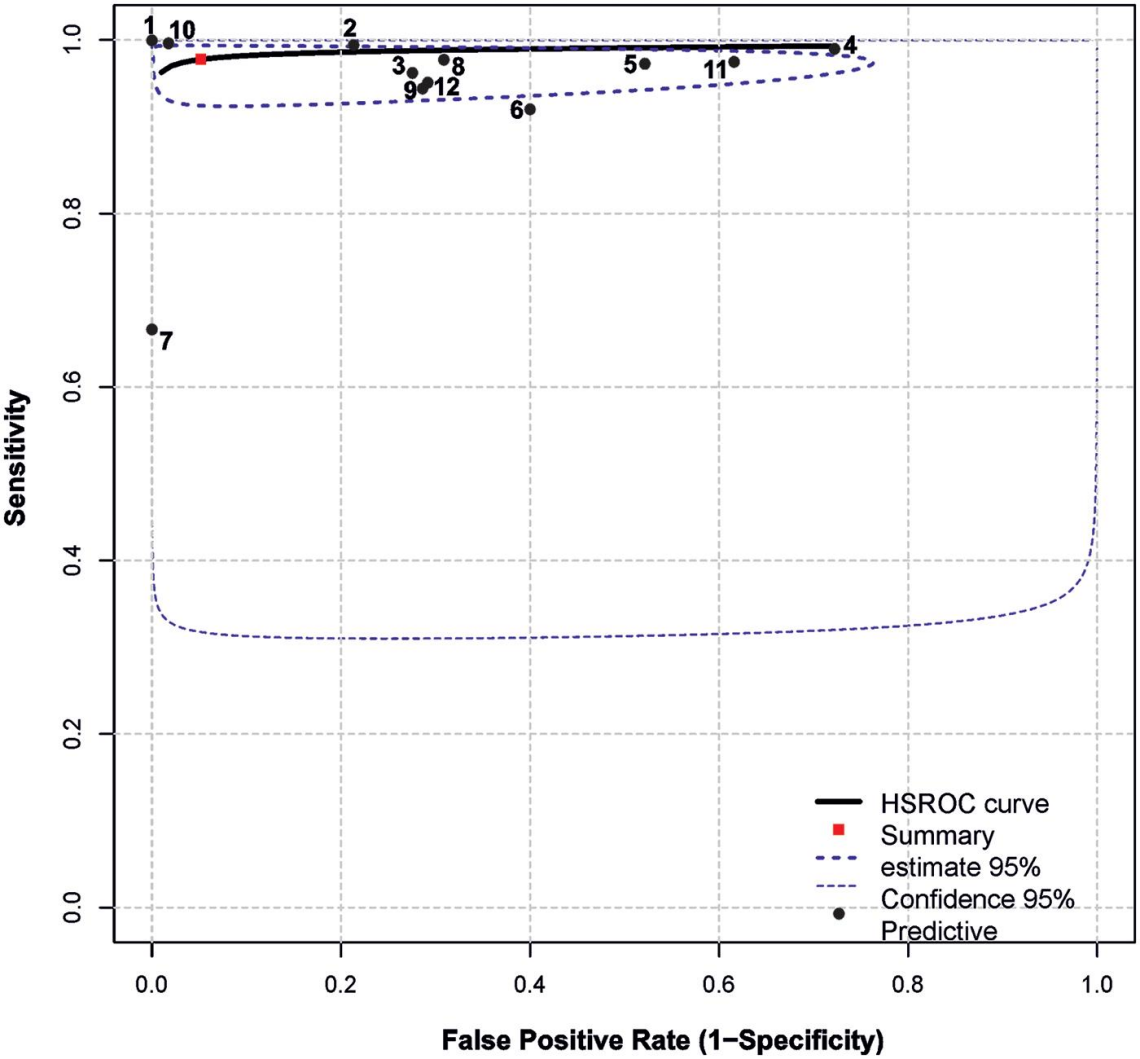
Summary ROC plot. The plot shows summary estimates, 95% confidence (dotted lines) and 95% prediction intervals (dashed lines). Designations: (1) Vasilev et al. (22); (2) Blake et al. (11); (3) AlJasmi et al. (28); (4) Plesner et al. (10); (5) Yoo et al. (24); (6) Schalekamp et al. (26); (7) Subramanian et al. (25); (8) Sridharan et al. (23); (9) Annarumma et al. (21); (10) Dyer et al. (27); (11) Nabulsi et al. (20)**(a)** (DS-1); (12) Nabulsi et al. (20)**(b)** CXR-14.

Summary results regarding the variability of key diagnostic metrics—sensitivity and specificity—are illustrated in the forest plots (Figure 2) and the ROC curve plot (Figure 3). Notably, substantial heterogeneity was observed in sensitivity estimates and especially in specificity estimates.

In most cases, relatively narrow confidence intervals were reported, which is typical for studies with large sample sizes. However, wide confidence intervals for diagnostic performance were observed in (26) and (20).

The highest simultaneous sensitivity and specificity values were reported in the population-based real-time study by (22) and the hospital-based dataset by (27).

Additionally, sensitivity values exceeding 95.0% were found in the studies by (10, 11, 20, 23, 24, 28), although these were accompanied by a substantial reduction in specificity, ranging from 28.0% to 79.0%.

In (26), sensitivity estimates demonstrated a relatively wide confidence interval and low specificity (60%). Conversely, a high specificity of 100.0% was observed in (25), albeit with a relatively low sensitivity of 67.0%.

Analysis of variability without factors revealed substantial between-study heterogeneity: $\tau^{2}$ (estimated amount of residual heterogeneity) = 0.0005, $I^{2}$ (residual heterogeneity/unaccounted variability) = 100.0%. The Q test for heterogeneity also indicated significant between-study heterogeneity (*p* < 0.01).

Incorporating of sample size as a factor into the model left the unexplained between-study heterogeneity high: *τ*^2^ = 0.0006, *I*^2^ = 100.0%, and $R^{2}$ (amount of heterogeneity accounted for) = 0.0%. The intercept was statistically significant in the model (0.97, *p* < 0.01), while the coefficient for the factor was not (0.0041, *p* = 0.93).

Including year of publication as a factor yielded a nonsignificant effect: *τ*^2^ = 0.0004, *I*^2^ = 22.5%, *R*^2^ = 20.2%. The intercept (−15.0617, *p* = 0.54) and factor coefficient (0.0079, *p* = 0.51) were both nonsignificant.

The population factor effectively explained the differences between studies: *τ*^2^ = 0.0, *I*^2^ = 0.0%, *R*^2^ = 100.0%. In the model, the intercept (0.96, *p* < 0.01) and factor coefficient (0.04, *p* < 0.01) were both statistically significant. The subgroup effect estimates for population were as follows: “general” subgroup — sensitivity 0.999 ± 0.0, specificity 1.0 ± 0.0; “hospital” subgroup — sensitivity 0.962 ± 0.0, specificity 0.7 ± 0.06.

### 3.4 Quality assessment

**Figure 4:**
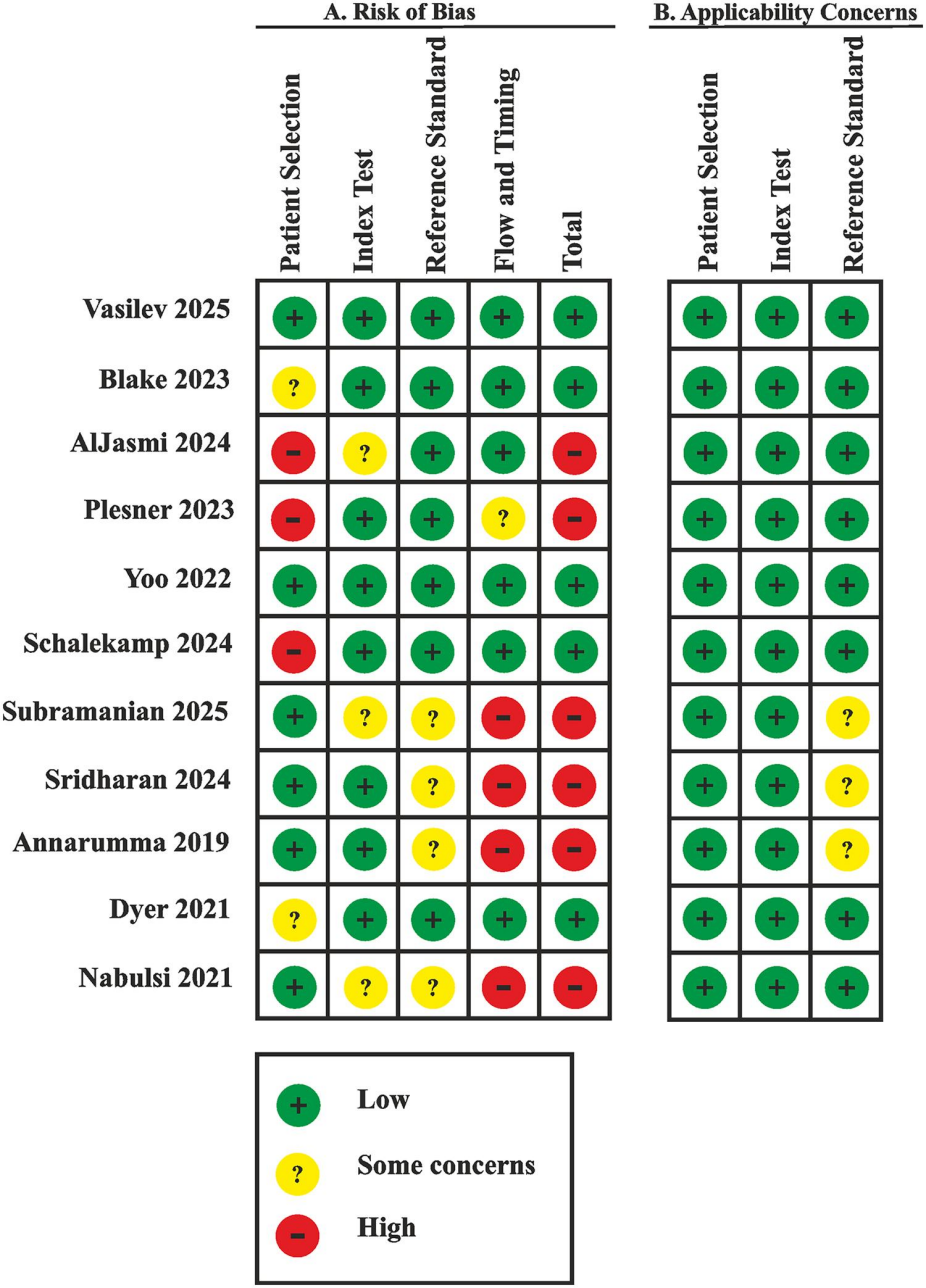
Risk of bias and applicability concerns summary: review authors' judgements about each domain for each included study.

The risk of bias assessment results according to QUADAS-CAD and QUADAS-2 is presented in Figure 4. A low risk of bias was assigned to six (55%) publications (11, 20, 22, 24, 26, 27). A high risk of bias was assigned to five (45%) publications (10, 21, 23, 25, 28).

The risk of bias in the Patient Selection domain was due only to a few screening studies using samples representative of the real-world population (22, 24, 25). In other cases, the sample composition was either biased (10, 26, 28) or there was a potential risk of bias (11, 20, 21, 23, 27).

A high risk of bias in the Flow and Timing domain was identified in three (27%) publications (21, 23, 25) due to concerns that not all cases employed the same reference standard and, correspondingly, due to unclear timing and transparency in obtaining results.

We calculated prevalence using the GRADE tool, based on the expected rate of CXRs observed in a real-world population (22) (45%), as well as minimum and maximum values.

The overall certainty of the evidence was rated as low owing to a high risk of bias affecting 45% of the included studies and the substantial heterogeneity of diagnostic estimates (Table 3), which was caused by significant differences in the analyzed populations.

**Table 3:** GRADEpro estimation: any CXR abnormalities. **Review question:** what is the accuracy of any CXR abnormality as a screening test for detecting abnormalities in a general population of people. **Role of index test:** to estimate number of autonomously sorting CXRs without abnormalities. **Reference standards:** radiologists reports. **Study design:** cross-sectional studies. Any CXR abnormality **summary sensitivity** (95% CI): 97.8% (94.8 to 99.1); **summary specificity** (95% CI): 94.8% (53.0 to 99.7).

Outcome	№ of studies (№ of patients)	Study design	Factors that may decrease certainty of evidence					Effect per 10 000 patients tested			Test accuracy CoE
			Risk of bias	Indirectness	Inconsistency	Imprecision	Publication bias	pre-test probability of 0.2%	pre-test probability of 45%	pre-test probability of 85%	
True positives (patients with normal/abnormal CXR)	11 studies 306,592 patients	cross-sectional (cohort type accuracy study)	serious^a^	not serious	serious^b^	not serious	none	20 (19 to 20)	4,401 (4,266 to 4,460)	8,313 (8,058 to 8,424)	⊕⊕○○ Low^a^^,^^b^
False negatives (patients incorrectly classified as not having normal/abnormal CXR)								0 (0 to 1)	99 (40 to 234)	187 (76 to 442)	
True negatives (patients without normal/abnormal CXR)	11 studies 26,37,945 patients	cross-sectional (cohort type accuracy study)	serious^a^	not serious	serious^c^	not serious	none	9,461 (5,289 to 9,950)	5,214 (2,915 to 5,484)	1,422 (795 to 1,496)	⊕⊕○○ Low^a^^,^^c^
False positives (patients incorrectly classified as having normal/abnormal CXR)								519 (30 to 4,691)	286 (16 to 2,585)	78 (4 to 705)	

**Explanations**.

a Downgraded by one since 5 of 11 studies (45%) have high risk of bias due Patient Selection domain (3 studies have high risk of bias and 5 studies have some concerns according risk of bias), Reference standard domain (3 studies have some concerns according risk of bias), and Flow and timing domain (3 studies have high risk of bias and 1 study has some concerns).

b Downgraded by one since high heterogeneity level of sensitivity and its CI estimation, variability in reference standards between studies and lack of clear levels in reference standards.

c Downgraded by one since the wide range of autonomously sorted results (15%–99.8%) that cannot be fully explained by inter-population variability.

### 3.5 Assessment of temporal and economic benefits, comparison of diagnostic accuracy results with physicians

The assessment of temporal benefits of automated triage compared to triage performed by physicians was conducted in one study (23). The study (23) reports that the mean difference between AI and radiologist turnaround time (defined as the interval from CXR upload to the system until approval of the radiology report by the radiologist) was 818.9 min, which was statistically significant (*p* < 0.05, Wilcoxon signed-rank test).

Economic efficiency was evaluated in a single study (22). The study (22) demonstrated economic viability, with costs reduced by 43.7% over a 5-month experimental period.

Diagnostic accuracy between AI and radiologists was compared in three studies (10, 20, 27). Overall, AI demonstrated superior diagnostic performance compared to radiologists.

In the (10), AI had significantly higher sensitivity for detecting abnormal CXRs than clinical radiology reports, with sensitivities of 99.1% vs. 72.3% respectively (*p* < 0.001).

The study (20) reported sensitivity value of 98.0% for AI and 48.0% for radiologists for the DS-1 dataset, with specificities of 38.0% and 96.0%, respectively. For the CXR-14 dataset, sensitivity was 95.0% for AI and 54.0% for radiologists, with specificity of 71.0% and 94.0%, respectively.

In the study by (27), the false negative (FN) rate for AI was 0.33%, which was substantially lower than the 13.5% FN rate observed for radiologists.

## 4 Discussion

The proportion of studies that are autonomously triaged varies widely across different publications (15.0%–99.8%) and is largely determined by whether the sample was obtained through screening a natural population or represents a biased cohort with an increased risk of pathology detection.

For studies conducted on samples reflecting the natural population (22, 24, 25), the proportion of autonomously triaged studies exceeds 40%, which closely aligns with the overall average of 42.3% across all publications. In the recent study by (22) involving real-world data and a large sample of over half a million cases, the proportion of autonomously triaged CXRs was found to be 54.8%. Consequently, this value can be considered a natural and practically expected benchmark. A subgroup analysis revealed that the differences between studies conducted in general and hospital populations accounted for a significant amount of the observed heterogeneity.

On average, AI algorithms demonstrated high sensitivity across studies: 97.8% (95% CI: 94.8–99.1%). The sensitivity level of radiologists when analyzing CXRs, which serves as a benchmark, ranges from 53.6% to 95.5% and is highly dependent on the radiologists' experience and the type of pathology (29, 30). In contrast to radiologists, AI algorithms can be finely tuned by adjusting the threshold to balance sensitivity and specificity. Theoretical research on a limited dataset has demonstrated the feasibility of tuning AI services to achieve 100% sensitivity while maintaining 77.4% specificity (31). In a prospective experimental study of 209,497 screening CXRs, the false-negative rate of the AI was significantly lower (0.04%) than that of radiologists (8.6%) (32). The AI also autonomously triaged 55.9% (117,041) of CXRs in this study. Maximizing sensitivity inevitably results in decreased specificity. Nevertheless, across the majority of analyzed studies, AI also exhibited an acceptable specificity level of 94.8% (95% CI: 53.0–99.7%). Direct comparisons of the sensitivity and specificity of AI vs. radiologists in the analyzed studies (10, 20, 27) show that the performance of AI screening was not inferior to that of radiologists.

The sensitivity analysis revealed no distortion of the estimated sensitivity and specificity values when extreme values were excluded from the overall sample or when studies that used prioritization instead of binarization were excluded.

Most studies used either small datasets (72.7%) or retrospective designs (81.8%), which contributed to heterogeneity and limited the quality of the evidence. Consequently, considerable interest is directed towards studies conducted in real-world settings (22, 23) with large datasets (22, 25, 28), especially those that satisfy both criteria (22). The high complexity of large-scale screening experiments under real-world conditions stems from the stringent regulatory environments in many countries (33).

Currently, only AI systems that are registered as medical devices can be integrated into clinical practice. Prospective studies only include a limited number of AI solutions, such as the ChestEye algorithm by Oxipit (34), LUNIT INSIGHT CXR by Lunit (23), and QXR by Qure.ai (28). However, large-scale studies addressing financing models and the integration of autonomous triage into national healthcare systems are lacking for these solutions.

The temporary benefits of autonomous triage are often evaluated indirectly. Many researchers report a reduced workload for radiologists, primarily based on the theoretical savings of resources derived from the proportion of CXRs that are autonomously triaged. A prospective study involving 20,944 CXRs assessed time savings, approximated as potentially freed radiologist time (77%) (23).

The actual economic impact has only been evaluated in one study (22), largely due to the legislative and infrastructural environment established by the Moscow Experiment — the largest global, prospective, multicenter study on the applicability, safety and quality of AI in real clinical settings (35–40). Economic efficiency calculations incorporated the costs of reporting and interpreting each CXR with and without autonomous triage, as well as the expenses involved in acquiring and maintaining the AI system. The proposed autonomous triage model yielded a 43.7% reduction in costs. Additionally, a protocol was proposed to eliminate clinically significant errors by implementing dual automated reviews, whereby two independent AI medical devices analyze each diagnostic image simultaneously, with the final decision favoring the patient. While this approach would double the interpretation fee, it is projected to maintain a 40.4% economic benefit.

Selective literature review indicates that AI is highly ready for the autonomous triage of screening CXRs due to its high diagnostic performance. This would enable a reduction in radiologists' workload, optimization of workflows, and budgetary savings. The proposed methodology can be scaled up to various AI solutions for CXR interpretation. The real-world experiment conducted on large datasets was successful (22). However, more studies in real clinical settings are needed to robustly assess the potential benefits and challenges of implementing this technology into practice.

Our review focuses on a specific aspect: the ability of modern AI to autonomously identify normal examinations during screening. The next step in determining the readiness of AI for clinical implementation in CXR analysis is to evaluate its ability to classify chest pathologies (41), detect single pathology (42, 43), and foreign objects (44). AI has already demonstrated impressive diagnostic performance in these areas.

This review has several limitations. We selected only the most comprehensive studies that included analyses of multiple pathologies with English abstracts. The number of such studies is rather limited. Furthermore, there is a lack of research conducted in real-world conditions.

## 5 Conclusions and practical recommendations

A systematic review of studies has demonstrated that modern AI systems enable safe autonomous triage of CXRs, allowing the identification of on average over 40% of normal examinations, with a mean sensitivity of up to 97.8%. In conclusion, the diagnostic performance of AI in the preventive screening of thoracic diseases and the autonomous sorting of CXRs is highly promising.

However, there is a need for large-scale studies based on real-world data, which to date have been reported only in limited publications. This limitation is associated with multiple evident legislative and infrastructural barriers that have been overcome in only a few countries.

The implementation of AI for autonomous triage has been demonstrated to optimize workflow processes and reduce the routine workload of radiologists. The implementation of an autonomous triage approach within public healthcare systems has been demonstrated to result in a substantial reduction in financial costs, with a reported decrease of 43.7%. The proposed double parallel autonomous triage model is expected to completely mitigate clinically significant errors.

## Data Availability

The original contributions presented in the study are included in the article/Supplementary Material, further inquiries can be directed to the corresponding author.

## Appendix 1 List of articles excluded during full-text screening

**Table:**

№	First Author/Year	Title/Journal	DOI	Reason for exclusion
1	Yoon 2024	Use of artificial intelligence in triaging of chest radiographs to reduce radiologists' workload/ European Radiology	10.1007/s00330-023-10124-1	Comparison of radiologists’ readings with and without AI assistance.
2	Pillai 2022	Multi-Label Chest X-Ray Classification via Deep Learning. /Journal of Intelligent Learning. Systems and Applications	10.4236/jilsa.2022.144004	Classification of individual pathologies by different AI systems.
3	Albahli 2021	AI-driven deep CNN approach for multi-label pathology classification using chest X-Rays/PeerJ Computer Science	10.7717/peerj-cs.495	Classification of individual pathologies by AI.
4	Karthik 2021	Performance of a deep learning CNN model for the automated detection of 13 common conditions on Chest x-rays/ TechRxiv	10.36227/techrxiv.16725727.v1	Classification of individual pathologies by AI.
5	Govindarajan 2022	Role of an Automated Deep Learning Algorithm for Reliable Screening of Abnormality in Chest Radiographs: A Prospective Multicenter Quality Improvement Study/Diagnostics	10.3390/diagnostics12112724	Classification of individual pathologies by AI.
