# Supplementary material for: Autonomous chest x-ray image classification, capabilities and prospects: rapid evidence assessment
Source: Front Digit Health. 2026 Jan 13;7:1685771. doi: 10.3389/fdgth.2025.1685771 (PMC12836300; doi:10.3389/fdgth.2025.1685771)
Supplement: Supplementary file 1 [file Datasheet1.pdf]

Autonomous Chest X-Ray Image Classification, Capabilities and Prospects: Rapid Evidence Assessment

Yuriy Vasilev, Alexander Bazhin, Roman Reshetnikov, Olga Nanova, Anton Vladzymyrskyy, Kirill Arzamasov, Pavel Gelezhe, Olga Omelyanskaya

Citation
Yuriy Vasilev, Alexander Bazhin, Roman Reshetnikov, Olga Nanova, Anton Vladzymyrskyy, Kirill Arzamasov, Pavel Gelezhe, Olga Omelyanskaya. Autonomous Chest X-Ray Image Classification, Capabilities and Prospects: Rapid Evidence Assessment. Not yet published.

PREVIEW

REVIEW TITLE AND BASIC DETAILS

Review title
Autonomous Chest X-Ray Image Classification, Capabilities and Prospects: Rapid Evidence Assessment

Condition or domain being studied
Adult Screening; Chest X-ray; Triage; Sensitivity

Rationale for the review
Chest radiography is one of the most frequently performed preventive radiological examinations. During screening, radiologists review and exclude a large number of normal CXRs. In order to mitigate the risk of missed pathologies and reduce the workload of radiologists, clinical decision support systems—particularly those employing artificial intelligence (AI)—have gained widespread adoption.It is important to assess the readiness of modern AI systems for implementation in routine screening practice.

Review objectives
The present study aimed to evaluate the capabilities of contemporary AI methods for the autonomous triage of CXRs and assess their prospects for integration into routine clinical practice.

To achieve this, it is essential to:

1. Assess the number of large-scale prospective screening studies focused on chest organs.

2. Evaluate the methodological quality of these studies and identify any potential risk of bias.

3. Determine the diagnostic performance levels of AI systems.

4. Analyze the economic benefits of implementing AI systems in real-world settings.

Keywords
autonomous triage; Screening; Chest X-ray; Artificial Intelligence; Radiology

Country
Russian Federation

ELIGIBILITY CRITERIA

Population

Included
Screening studies that used CXRs of adults to analyze multiple pathologies.

Excluded

1. Single-pathology screening studies.
2. Diagnosis of pathologies from a limited set of pathological conditions.
3. Comparison of radiologists' readings with and without AI assistance.
4. Adolescents people - individuals under 18 years of age.

Intervention(s) or exposure(s)

Included
Triage

Automated screening using AI to exclude CXRs with a high likelihood of being normal.bsence of interventions or exposures.

Excluded
Case reports and reviews

Comparator(s) or control(s)

This review does not have any comparators

Study design

Both randomized and nonrandomized study types will be included.

Included
Observational, non-randomised trials, and randomised trials.

Excluded
Case reports.

Context

General populations are preferred. Screening studies conducted on hospital populations will also be included.

Studies from high-, middle-, and low-income countries will be included.

Screening studies employing both binarization and prioritization will be considered.

SIMILAR REVIEWS

Check for similar records already in PROSPERO

PROSPERO identified a number of existing PROSPERO records that were similar to this one (last check made on 1 December 2025). These are shown below along with the reasons given by that the review team for the reviews being different and/or proceeding.

- "AI-Driven Triage: A Meta-Analysis on Enhancing Accuracy and Efficiency in Emergency Departments" [published 20 August 2025] [CRD420251125909]. The review was judged **not to be similar**
- Application of Artificial Intelligence Models Based on Medical Imaging in Dental Caries Diagnosis: A Systematic Review and Meta-Analysis [published 21 July 2025] [CRD420251108203]. The review was judged **not to be similar**
- Artificial Intelligence in Ophthalmic Diagnosis and Treatment: A Systematic Review [published 29 November 2025] [CRD420251241901]. The review was judged **not to be similar**

TIMELINE OF THE REVIEW

Date of first submission to PROSPERO

This record has not been submitted.

Review timeline

Start date: 22 April 2025. End date: 30 December 2025.

Date of registration in PROSPERO

This record has not been published.

AVAILABILITY OF FULL PROTOCOL

Availability of full protocol
No preview available

SEARCHING AND SCREENING

Search for unpublished studies
Only published studies will be sought.

Main bibliographic databases that will be searched
The main database to be searched is *PubMed*.

Other important or specialist databases that will be searched
arXiv, medRxiv, and Elibrary

Search language restrictions
The review will only include studies published in English.

Search date restrictions
Databases will be searched for articles published from 1 January 2019, there are no search end date restrictions.

Other methods of identifying studies
Other studies will be identified by: *contacting authors or experts*, *looking through all the articles that cite the papers included in the review ("snowballing" or forward citation searching)*, *reference list checking (backward citation searching)*, *searching conference proceedings* and *searching dissertation and thesis databases*.

Additional information about identifying studies
Additional literature search using Google Scholar web search engine.

Link to search strategy
No preview available

Selection process
Studies will be screened independently by at least two people (or person/machine combination) with a process to resolve differences.

Other relevant information about searching and screening
The search query used in PubMed will be as follows:

("Radiography, Thoracic"[Mesh] OR Thoracic Radiograph*[tiab] OR (x-ray[tiab] AND (chest[tiab] OR thora*[tiab])) OR CXR) AND ("Radiographic Image Interpretation, Computer-Assisted"[Mesh] OR Artificial Intelligence[tiab] OR Computer vision[tiab] OR (learning[tiab] AND (machine[tiab] OR deep[tiab]))) AND (Autonom*[tiab] OR standalone[tiab] OR unaided[tiab] OR unassisted[tiab]).

The search query for arXiv will be: "Autonomous AI AND chest X-ray OR CXR AND triage". The search query for medRxiv, Elibrary, and Google Scholar was: "Autonomous AI chest X-ray triage".

DATA COLLECTION PROCESS

Data extraction from published articles and reports
Data will be extracted independently by at least two people (or person/machine combination) with a process to resolve differences.

Authors will be asked to provide any required data not available in published reports.

Study risk of bias or quality assessment
Risk of bias will be assessed using: *QUADAS-2*

QUADAS-CAD

Data will be assessed by one person (or a machine) and checked by at least one other person (or machine).

Additional information will **not** be sought from study investigators if required information is unclear or unavailable in the study publications/reports.

Reporting bias assessment

Risk of bias due to missing results will not be assessed

Certainty assessment

GRADE methodology will be used to assess the certainty of the evidence.

OUTCOMES TO BE ANALYSED

Main outcomes

Sensitivity and specificity of AI.

Additional outcomes

1. Values of true positives (TP), false negatives (FN), false positives (FP), true negatives (TN).

2. Total number of CXRs, number (%) of autonomously sorted CXRs

3. Any assessment of time benefits and economic efficiency.

PLANNED DATA SYNTHESIS

Strategy for data synthesis

1. The meta-analysis will be conducted using the MetaDTA tool (doi: 10.1002/jrsm.143944) including forest plots and HROC analyses.

2. Subgroup and random effects analyses will be conducted with the metafor package for R.

3. Sensitivity analysis conducted with MetaDTA.

CURRENT REVIEW STAGE

Stage of the review at this submission

Review stage	Started	Completed
Pilot work	✓	✓
Formal searching/study identification	✓	✓
Screening search results against inclusion criteria	✓	✓
Data extraction or receipt of IPD	✓	✓
Risk of bias/quality assessment	✓	✓
Data synthesis	✓	✓

Review status

The review is completed.

Publication of review results

Results of the review will be published in English.

REVIEW AFFILIATION, FUNDING AND PEER REVIEW

Review team members

Dr Yuriy Vasilev. ORCID: 0000-0002-5283-5961. Research and Practical Clinical Center for Diagnostics and Telemedicine Technologies of the Moscow Health Care Department, Moscow, Russia. Russian Federation.

No conflict of interest declared.

Dr Alexander Bazhin. ORCID: 0000-0003-3198-1334. Research and Practical Clinical Center for Diagnostics and Telemedicine Technologies of the Moscow Health Care Department, Moscow, Russia. Russian Federation.

No conflict of interest declared.

Dr Roman Reshetnikov. ORCID: 0000-0002-9661-0254. Research and Practical Clinical Center for Diagnostics and Telemedicine Technologies of the Moscow Health Care Department, Moscow, Russia. Russian Federation.

No conflict of interest declared.

Dr Olga Nanova (review guarantor and contact) ORCID: 0000-0001-8886-3684. Research and Practical Clinical Center for Diagnostics and Telemedicine Technologies of the Moscow Health Care Department, Moscow, Russia. Russian Federation.

No conflict of interest declared.

Professor Anton Vladzymyrskyy. ORCID: 0000-0002-2990-7736. Research and Practical Clinical Center for Diagnostics and Telemedicine Technologies of the Moscow Health Care Department, Moscow, Russia. Russian Federation.

No conflict of interest declared.

Dr Kirill Arzamasov. ORCID: 0000-0001-7786-0349. Research and Practical Clinical Center for Diagnostics and Telemedicine Technologies of the Moscow Health Care Department, Moscow, Russia. Russian Federation.

No conflict of interest declared.

Dr Pavel Gelezhe. ORCID: 0000-0003-1072-2202. Research and Practical Clinical Center for Diagnostics and Telemedicine Technologies of the Moscow Health Care Department, Moscow, Russia. Russian Federation.

No conflict of interest declared.

Olga Omelyanskaya. ORCID: 0000-0002-0245-4431. Research and Practical Clinical Center for Diagnostics and Telemedicine Technologies of the Moscow Health Care Department, Moscow, Russia. Russian Federation.

No conflict of interest declared.

Named contact

Dr Olga Nanova (nanovaolgag@gmail.com). ORCID: 0000-0001-8886-3684. Research and Practical Clinical Center for Diagnostics and Telemedicine Technologies of the Moscow Health Care Department, Moscow, Russia. Russian Federation.

Review affiliation

Research and Practical Clinical Center for Diagnostics and Telemedicine Technologies of the Moscow Health Care Department, Moscow, Russia

Funding source

Grant number
125051305989-8

Additional non-commercial funding information
Moscow Government Grant

Additional information about funding
This article will be prepared by a team of authors within the framework of a scientific and practical project in the field of medicine (No. EGISU: 125051305989-8) "A promising automated workplace of a radiologist based on generative artificial intelligence".

Peer review

There has been no peer review of this planned review.

ADDITIONAL INFORMATION

Review conflict of interest

Declared individual interests are recorded under team member details.. No additional interests are recorded for this review.

Medical Subject Headings

No preview available

PROSPERO version history

No preview available

Disclaimer

The content of this record displays the information provided by the review team. PROSPERO does not peer review registration records or endorse their content.

PROSPERO accepts and posts the information provided in good faith; responsibility for record content rests with the review team. The guarantor for this record has affirmed that the information provided is truthful and that they understand that deliberate provision of inaccurate information may be construed as scientific misconduct.

PROSPERO does not accept any liability for the content provided in this record or for its use. Readers use the information provided in this record at their own risk.

Any enquiries about the record should be referred to the named review contact
